# Radiothermometric Study of the Effect of Amino Acid Mutation on the Characteristics of the Enzymatic System

**DOI:** 10.3390/diagnostics12040943

**Published:** 2022-04-10

**Authors:** Yuri D. Ivanov, Kristina A. Malsagova, Natalia S. Bukharina, Sergey G. Vesnin, Sergey A. Usanov, Vadim Yu. Tatur, Andrei A. Lukyanitsa, Nina D. Ivanova, Vladimir A. Konev, Vadim S. Ziborov

**Affiliations:** 1 Laboratory of Nanobiotechnology, Institute of Biomedical Chemistry, Pogodinskaya St. 10 Build. 8, 119121 Moscow, Russia; natalia.bukho@gmail.com (N.S.B.); ziborov.vs@yandex.ru (V.S.Z.); 2 Laboratory of Shock Wave Impacts, Joint Institute for High Temperatures of Russian Academy of Sciences, Izhorskaya St. 13 Build. 2, 125412 Moscow, Russia; 3 RES Ltd., Bolshaya Pochtovaya St. 22, 105082 Moscow, Russia; vesnin47@gmail.com or; 4 Institute of Bioorganic Chemistry, National Academy of Sciences of Belarus, Academician V.F. Kuprevich 5 Build. 2, 220141 Minsk, Belarus; usanov@iboch.bas-net.by; 5 Foundation of Perspective Technologies and Novations, Shipilovskaya St. 64, 115682 Moscow, Russia; tatur@mail.ru (V.Y.T.); andrei_luk@mail.ru (A.A.L.); 6 Skryabin Moscow State Academy of Veterinary Medicine and Biotechnology, Academician Skryabin St. 23, 109472 Moscow, Russia; nina72.ivaniva72@gmail.com; 7 Department of Infectious Diseases in Children, Faculty of Pediatrics, Pirogov Russian National Research Medical University, Ostrovityanov St. 1, 117997 Moscow, Russia; konev60@mail.ru

**Keywords:** radiothermometry, cytochrome, CYP102 A1, brightness temperature

## Abstract

The radiothermometry (RTM) study of a cytochrome-containing system (CYP102 A1) has been conducted in order to demonstrate the applicability of RTM for monitoring changes in the functional activity of an enzyme in case of its point mutation. The study has been performed with the example of the wild-type cytochrome (WT) and its mutant type A264K. CYP102 A1 is a nanoscale protein-enzymatic system of about 10 nm in size. RTM uses a radio detector and can record the corresponding brightness temperature (*T_br_*) of the nanoscale enzyme solution within the 3.4–4.2 GHz frequency range during enzyme functioning. It was found that the enzymatic reaction during the lauric acid hydroxylation at the wild-type CYP102 A1 (WT) concentration of ~10^−9^ M is accompanied by *T_br_* fluctuations of ~0.5–1 °C. At the same time, no *T_br_* fluctuations are observed for the mutated forms of the enzyme CYP102 A1 (A264K), where one amino acid was replaced. We know that the activity of CYP102 A1 (WT) is ~4 orders of magnitude higher than that of CYP102 A1 (A264K). We therefore concluded that the disappearance of the fluctuation of *T_br_* CYP102 A1 (A264K) is associated with a decrease in the activity of the enzyme. This effect can be used to develop new methods for testing the activity of the enzyme that do not require additional labels and expensive equipment, in comparison with calorimetry and spectral methods. The RTM is beginning to find application in the diagnosis of oncological diseases and for the analysis of biochemical processes.

## 1. Introduction

Radio biology and radio medicine is a field of science that develops the theory and practice of application of radiation for biology and medical purposes [1,2,3,4,5,6,7,8,9,10,11,12,13]. The radiothermometry method is relatively inexpensive and allows one to detect radiation in the microwave range in real-time mode without using additional tags. The RTM method is based on monitoring the *T_br_*, which can change during the biochemical reaction [9]. The *T_br_* is a temperature value that equals the thermodynamical temperature of a complete radiator.

During the biochemical reaction, a non-balanced condition of the medium can appear that is characterized with the increase in *T,* which can be accompanied by radiation in a certain frequency range. This is why the radio detector used allows us not only to conduct diagnostics to detect tumors on a macrolevel, but also to measure the kinetics of biochemical processes on a milli-level (tumours [3,4,5,6,7,8]), micro-level (cell processes [10]) and even on a nano-level (enzyme reactions, denaturation processes [11,12]) in a microwave range to monitor the changes in *T_br_*.

Regarding the milli-level, the use of RTM for the diagnosis of socially significant diseases was reported in a number of papers: the revelation of cancer at an early stage by detecting an increase in local brightness temperature (in microwave range) in the tumour growth region was reported [1,2,3,4,5,6,7,8]. In addition to standard methods, such as X-ray diagnostics, the RTM method can provide additional “energetic” information on the intensity of proliferative processes and the speed of the tumor growth, etc. It was noted that combining mammography and the RTM method lowers the risk of false negative results three- or four-fold and raises the diagnostics’ sensitivity up to 98% [3].

Regarding micro- and nano-level, studies on the use of radiation occurring during the modulation of functioning of enzymes, either with or without the introduction of microparticle labels, were reported. So, as an example of an approach utilizing labels, the application of micron-sized ferromagnetic iron-oxides for local thermal control of amylase activity with the use of 0.34 MHz radio frequency range can be pointed out [14].

Magnetic nanoparticles of 6 nm to 70 nm in diameter can be employed for the modulation of activity of β-galactosidase, bovine carbonic anhydrase and thermolysin [15,16,17]. An increase in protease activity after the irradiation of the enzyme, labeled with 4 nm gold-coated magnetite particles in a radio frequency (17.76 MHz) field, owing to a conversion of the radio frequency radiation into local heat was demonstrated [18]. Moreover, a very interesting approach to the local regulation of the properties of proteins with the use of iron oxide nanoparticles of various sizes was recently reported by Ovejero et al. [19]. These authors developed a selective magnetic nanoheating approach for the multi-hot-spot induction and sequential regulation of enzymes [19]. This approach creates a new paradigm, providing an opportunity for the selective regulation of multi-enzyme reactions.

Within the range of shorter microwaves, modulation of the properties of proteins was demonstrated even without the introduction of nanometer-size particles. So, electromagnetic irradiation was demonstrated to induce conformational transitions in hemoglobin [20]. The possibility of conformational changes in macromolecules upon the impact of electromagnetic radiation was shown with the example of antibody/antigen interaction [21].

On a nanoscale, studies on the registration of self-radiation of label-free enzyme systems are important. It is known that nanoscale heme-containing enzymatic systems based on horseradish peroxidase (HRP) and CYP102 A1, where enzymatic components have the size of about 10 nm, as noted above [22], can emit in the microwave band as they function [9,12,23]. In these studies, radiation was detected using the RTM method in the microwave range of 3.4 to 4.2 GHz. The question is, however, whether or not there can be a change in the microwave radiation of enzyme systems upon point mutations in the enzymes. This is particularly important, since the increased expression of the mutant forms of proteins is known to take place in some cases of oncological diseases [13,24,25]. We should note that cytochromes P450 and their mutant forms can take part in cancer formation and cancer treatment. They mediate metabolic activation of numerous pre-carcinogens and participate in the activation and inactivation of anti-tumor medicines [26]. This makes developing and applying new methods to analyze the functional condition of these systems in order to receive fuller, more relevant information about them.

To study the mechanism of cytochrome P450 functioning, a simpler model bacterial system CYP102A1 of the cytochrome P450 enzyme superfamily characterized in [22] is usually employed, and, in the present study, we have also used this model system. CYP102A1 represents a self-sufficient enzyme, where reductase and heme domains are linked in one polypeptide chain [23]. The latter fact raises interest in it as a convenient simplified model of a transport chain of monooxygenase systems containing cytochrome P450, which plays a role in CYP102A1 catalysis of hydroxylation of saturated and unsaturated fatty acids [27]. As we demonstrated previously, during the functioning of an enzyme system containing 10^−9^ M of the CYP102 A1 enzyme, microwave radiation has been observed in the form of *T_br_* pulses [9].

In the present work, the registration of the influence of the effect of a point mutation in cytochrome P450 CYP102A1 on the brightness temperature in a model reconstructed system. For this purpose, we compared *T_br_* fluctuation for the wild type of CYP102 A1 (WT) and its mutant type CYP102 A1 (A264K). The activity of CYP102 A1 (WT) in the presence of the lauric acid (LA) is *k_cat_* = 50 s^−1^ at the enzyme concentration of ~10^−9^ M [28]. In addition to this, we know that the activity of the mutant type CYP102 A1 (A264K) upon lauric acid hydroxylation is about *k_cat_* = 0.006 s^−1^ [29], which is three orders lower than the activity exhibited by CYP102 A1 (WT). Therefore, we have chosen CYP102 A1 (A264K) for our study in order to conduct a comparative analysis of radiation of solutions in the presence of a significantly less active CYP102 A1, but at the same enzyme concentration. We have shown that the passage from the wild type of protein to its mutant type connected with an 8333-fold increase in activity leads to the disappearance of microwave radiation. Thus, we have shown that the *T_br_* fluctuation of the solution CYP102 A1 is dependent on its activity.

## 2. Materials and Methods

### 2.1. Chemical Agents

Phosphate-buffered saline (2 mM, pH 7.4) containing 75 mM NaCl was purchased from Pierce (Waltham, MA, USA). Deionized ultrapure water was obtained using a Simplicity UV system (Millipore, Molsheim, France). Sodium laurate and NADPH were purchased from Sigma (St. Louis, MO, USA).

### 2.2. Proteins

Wild type protein cytochrome CYP102 A1 (WT) was provided by Professor S.A. Usanov and expressed according to [28]. The sample protein solutions (~10^−9^ M) were prepared from the stock solution (50 µM in 23 mM potassium phosphate buffer) with the use of subsequent ten-fold dilution in the in-process buffer. Mutant type protein CYP102 A1 (A264K) was kindly provided by the laboratory of Professor V.G. Zgoda (Institute of Biomedical Chemistry, Moscow, Russia) and prepared according to [30].

### 2.3. Analytical Measurements

Protein concentration was determined using the spectrophotometry method. CYP102 A1 (WT) and (A2643K) absorption spectra were measured using spectrophotometer Agilent Model 8453 at the temperature of 23 °C. The pure CYP102 A1 (WT) and (A2643K) concentrations were determined from the differential absorption spectra of carboxycomplex of their recovered forms, using the extinction coefficient of 91 mM^−1^ cm^−1^ for the absorption difference at wavelengths of 450 nm and 490 nm, according to the method described in [31].

### 2.4. Methods of Monitoring Microwave Radiation of CYP102 A1 Solution

#### 2.4.1. Catalytic Reaction in CYP102 A1 System

Catalytic reaction in the enzymatic system has been conducted in a measurement cell with a reconstructed (WT) or (A2643K) system containing CYP102 A1 and its substrate, lauric acid, LA (0.5 mM) in PBS-D, following a similar scheme as described in [9]. The reaction was initiated by adding a water solution NADPH (0.2 mM) into the incubating medium. Measurement conditions: volume of sample solution 200 µL, temperature 23 °C. The check measurements of the solution brightness temperature were taken using two solution types: the one not containing a substrate or the one not containing electron donor NADPH. The duration of measurements was at least 400 s.

#### 2.4.2. Measuring Microwave Radiation of CYP102 A1 Solution

As a microwave detector, we used a broadband radiometer RTM-01 RES, operating range: 3.4 to 4.2 GHz. In order to measure the solution’s *T_br_*, we completely immersed the detector’s buggy-whip antenna into the sample solution similar to the one in [9].

As we noted in the introduction, the radiometer measures the *T_br_* of the object. Radiation power, *P* can be assessed in the frequency range Δ*f* measured by the radiometer.

We know that

*P* = *kT*Δ*f*, (1)

where *k* is the Boltzmann constant, *T* is the temperature, and Δ*f* is the frequency range [32]. Equation (1) shows that the radiation power is proportional to *T_br_*. Therefore, from here on the radiation power (radiation energy for the frequency band Δ*f*) will be measured in degrees. We will thus use *T_br_* units that the radiometer RTM-01 RES is divided into to show the results we acquired. Our measurements shall be ±0.1 °C precise. These measurements figures shall be shown as a brightness temperature’s function of time, *T_br_*(*t*).

### 2.5. AFM Visualization

#### 2.5.1. Sample Preparation

First, 0.5 mM CYP102A1 in 10 mM phosphate-buffered saline (pH 7.4) was applied to a mica AFM chip. Then, the AFM chip was washed with distilled water and placed in 2.5 mM phosphate-buffered saline. The measurements were carried out at an air temperature of ~25 °C.

#### 2.5.2. Atomic force Microscopy Measurements

Visualization of CYP102A1 molecules was carried out employing an atomic force microscope (AFM Dimension 3100, Digital Instruments, Veeco) in liquid. The measurements were conducted in a semi-contact mode using DNP-S10 probes (Veeco). The stiffness constant of the probes was 0.32 ÷ 0.58 N/m. The AFM scanning was performed in semi-contact mode, analyzing the time dependence of the height fluctuations of CYP102A1 molecules according to the method described in [30,33].

### 2.6. AFM Data Processing

The processing of AFM data and measurement of the heights of the visualized objects were conducted using the AFM dtp software (IBMC, Russian Academy of Medical Sciences, Moscow, Russia). The heights of CYP102A1 protein molecules were determined as the corresponding maxima of their height distribution *ρ*(*h*) using the following equation:

(2) $$\rho (h)=\frac{N_{h}}{N}*100\%$$

where *N_h_* is the number of proteins visualized with height *h*, and *N* is the total number of proteins visualized. The experimental dependence (1) was approximated using the Gaussian function:

(3) ρ(h)=∑ρi(h)=∑i=12Ae−4ln(2)(h−hc)2w2wπ4ln(2)

where *A*, *h_c_*, *w* are parameters varied during approximation. In this case, the position of the maximum height distribution of objects was calculated as the maximum of the approximating function (2) for each distribution. The curve was approximated by the sum of two exponentials, and the obtained AFM images of visualized objects were divided into two groups with the corresponding maxima of *h_max_*_1_ and *h_max_*_2_. 

### 2.7. Analysis of CYP102A1 Molecules’ Activity Using the AFM Method

The analysis of the enzyme molecules’ activity was carried out similarly to the method described in [33]. This method has been adapted for both (WT) and (A264K) CYP102A1 molecules in [30]. Under this method, an increase in the oscillation amplitude of the CYP102A1 enzyme globule in the process of substrate hydroxylation is measured. This approach was used in the present work to analyze the activity of the CYP102A1 protein both (WT) and (A264K).

For this purpose, after AFM visualization of the enzyme, the height fluctuations of the CYP102A1 enzyme oligomers both (WT) and (A264K) were monitored during the reaction of lauryl acid hydroxylation. AFM measurements of the height fluctuations of single CYP102A1 molecules were carried out as described above (Section 2.6 in Materials and Methods) according to the techniques described elsewhere [30,33]. In order to do so, the scanning area was chosen so that the selected enzyme molecule could be found in the AFM frame. Then, having selected this molecule, scanning along the slow axis was turned off. By doing so, a time sweep image of the same part of the molecule was obtained. The scanning frequency was set to 1 Hz. In each image, the rms value of the height fluctuations of the enzyme molecule was calculated as the rms value of the fluctuations of the molecule vertex, minus the background fluctuations from the surface of the AFM chip.

The height fluctuations (∆*h*) of the CYP102A1 enzyme molecule oligomers, both (WT) and (A264K), were obtained in 2.5 mM PBS-D buffer in the presence of its substrate, lauric acid (0.5 mM) in the absence of NADPH (inactive oxidated coditions), and in the presence of both the substrate and NADPH (0.2 mM) (hydroxylation reaction conditions).

## 3. Results

### 3.1. Results of Microwave Radiation Check-Measurements

**Figure 1:**
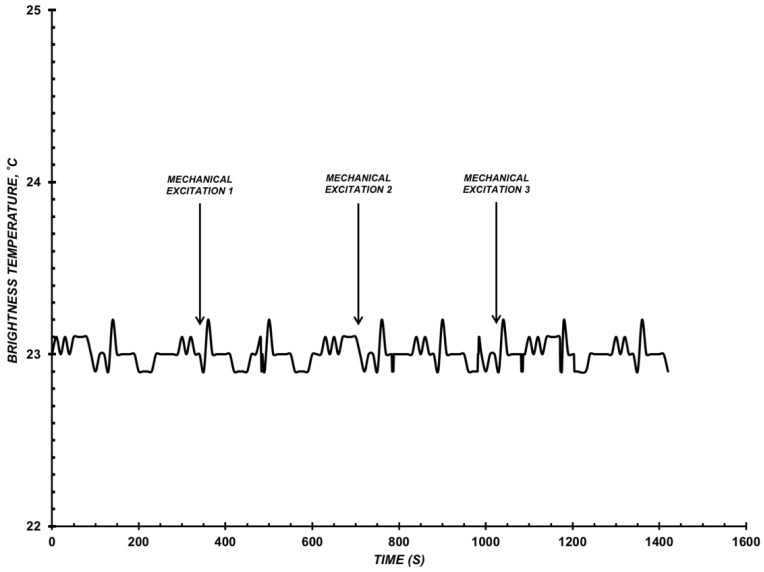
Example of the influence of mechanical excitation on *T_br_*(*t*). Assay conditions: inactive reconstructed CYP102 A1 system, an incubation mixture in the cell: C_CYP102A1_ = 10^−9^ M, 0.5 mM LA, 10 mM phosphate-buffered saline, pH 7.2, V = 200 µL, T_solution_ = 23 °C. Arrows indicate the excitation of the solution in the cell.

The solution containing CYP102 A1 enzyme (WT or its mutant type A264K), as well as its substrate LA, but not containing NADPH, is a reconstructed cytochrome P450BM3-containing system in its inactive state. Two variants of check-experiments were carried out:

(1) Experiments were held in order to find out the basic level of microwave radiation noises that emerge during mechanical excitation in an inactive enzyme CYP102 A1 system. For this, we mixed a solution containing protein and its substrate, the lauric acid, but not containing electron donor NADPH. 

(2) Experiments were held in order to detect how adding NADPH into the buffer solution affects the microwave radiation level. For this purpose, NADPH was added into a solution containing CYP102 A1 WT or A264K but without a substrate. The results of type 1 check-experiments have shown that a slight increase in *T_br_* is observed at the mechanical excitation of the solution. This increase value was further used as the noise level of the microwave radiation of the solution containing CYP102 A1 (WT) protein at the concentration of ~10^−9^ M and LA, but not containing NADPH (Figure 1). The detected noise level of the microwave radiation did not exceed ±0.5 °C. As an example, Figure 1 shows typical measurement results as per type 2 in the presence of CYP102 A1 (WT) and (264K), where we can see the emergence of such a noise impulse after adding NADPH or when stirring the solution.

**Figure 2:**
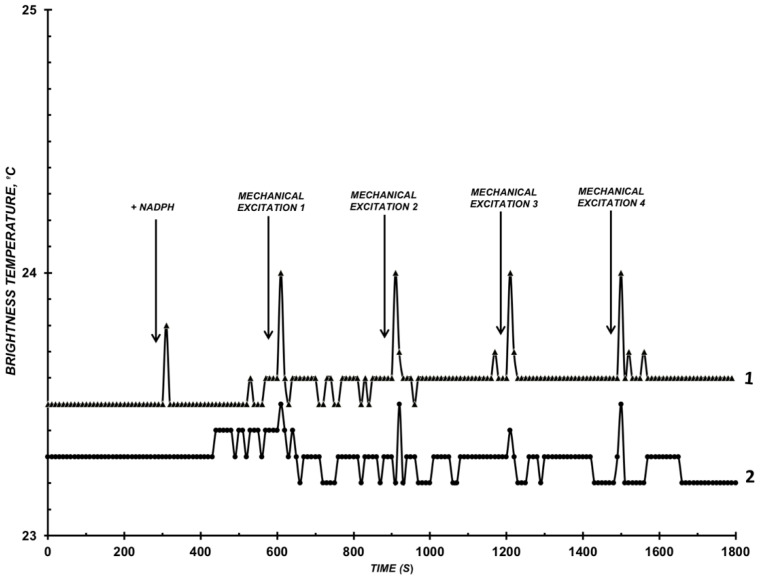
Example of how adding NADPH into the buffer influences *T_br_*(*t*). Assay conditions: incubation mixture in the cell: C_CYP102A1_ = 10^−9^ M, 10 mM phosphate-buffered saline, pH 7.2, V = 200 µL, T_solution_ = 23 °C. Curve 1 (triangles)—CYP102 A1 (WT); curve 2 (circles)—CYP102 A1 (264K). Arrows indicate adding electron donor NADPH into the system and moments of stirring the solution in the cell.

When conducting check-experiments as per the variant 2, the influence of adding NADPH into the system on the microwave radiation levels was observed (Figure 2).

As shown in Figure 2, changes in the level of microwave radiation of the solution did not exceed ±0.5 °C when adding NADPH into the system containing CYP102 A1 (WT) or (264K), but not containing the substrate. 

### 3.2. Results of Detecting Microwave Radiation of CYP102 A1 WT and A264K Solution

Microwave radiation of CYP102 A1 (WT) enzyme and its mutant type (A264K) in the process of catalytic cycle was detected at the temperature of 23 °C. Earlier, at this temperature, we obtained results on the radiation of enzyme solutions of wild type CYP102 A1 (WT) in the presence of LA substrate. 

**Figure 3:**
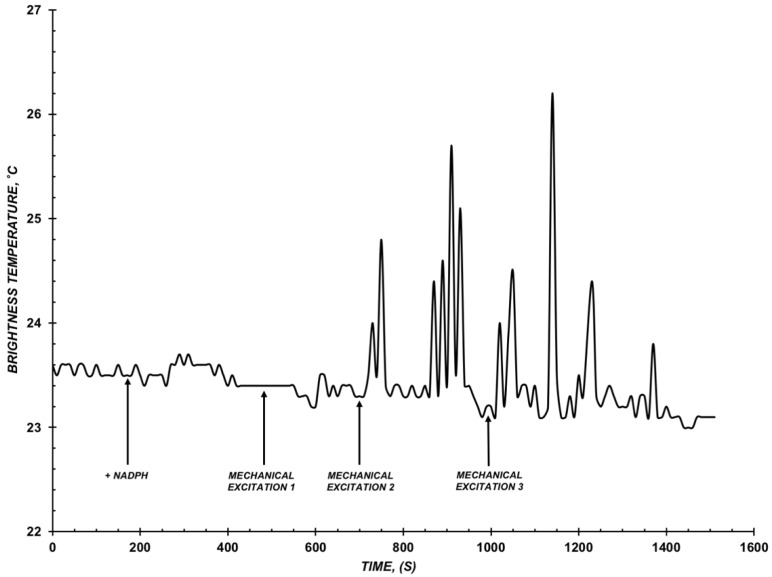
Example of *T_br_*(*t*) dependence of the reconstructed CYP102 A1 (WT) system. Assay conditions: C_CYP102A1_ = 10^−9^ M, 0.5 mM LA, 10 mM phosphate-buffered saline, pH 7.2, V = 200 µL, T_solution_ = 23 °C. Arrows indicate adding the electron donor NADPH into the system and moments of stirring the solution in the cell.

Radiation processes have been studied when using CYP102 A1 (WT) at the concentration of ~10^−9^ M. For this concentration, radiation was detected that was expressed through the emergence of impulses on the curve of function of *T_br_* of the sample solution when conducting an enzymatic reaction. An example of obtained measurements data of the *T_br_*(*t*) dependence is shown in Figure 3.

As shown in Figure 3, when NADPH is added into the solution containing protein at the concentration of 10^−9^ M and the substrate, significant changes are observed in the *T_br_*(*t*) dependence as a pulse train. At the same time, the total number of pulses over the entire observation period was about 8. The interval of intensity of pulses was ~0.8 to 1.9 °C, whereas their length was 20 to 40 s.

Pulse trains emerged with a delay after initiating the enzymatic reaction by adding NADPH (for approximately 500 s) and were observed after the mechanical excitation 2 at the 90th second (Figure 3). The total energy output of the radiation over the entire time of the observation was about 15.7 °C.

**Figure 4:**
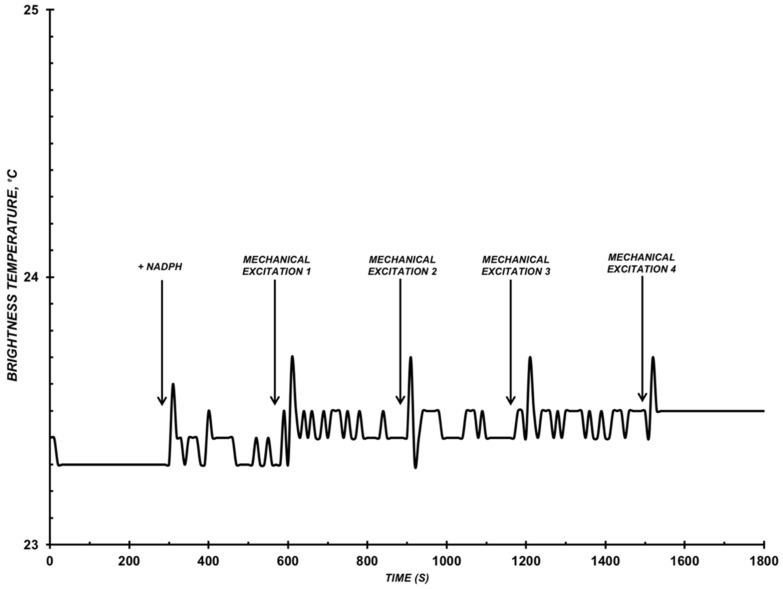
Example of *T_br_*(*t*) function of the reconstructed CYP102 A1 (264K) system. Assay conditions: C_CYP102A1_ = 10^−9^ M, 0.5 mM LA, 10 mM phosphate-buffered saline, pH 7.2, V = 200 µL, T_solution_ = 23 °C. Arrows indicate adding electron donor NADPH into the system and moments of stirring the solution in the cell.

Then, assays with the mutant type of CYP102 A1 (A264K) enzyme in the presence of LA were conducted (Figure 4).

We know that the activity of this enzyme is over 3 orders lower than the one of its wild type, as indicated above in the Introduction. No radiation was registered for this system (Figure 4). The standard deviation in the experiments with the mutant enzyme did not exceed 0.5 °C.

## 4. Discussion

In the CYP102 A1 enzyme system, the aqueous medium represents a heterogeneous structure containing para- and ortho-isomers of water, while the entire enzyme system is a more complex organization of the medium, where a protein is surrounded by an ice-like water shell of para-isomers of water [34]. Monooxygenases catalyze reactions of integration of one oxygen atom into different substrates, the other oxygen atom recovers to water. The general scheme of catalytic mechanism can be presented as follows [35]:

RH + O_2_ + 2e^−^ + 2H^+^ → ROH + H_2_O,

where RH is the substrate (LA), and (2e^−^ + 2H^+^) is the electron donor, which can be both NADPH (in the case of CYP102A1). In the process of functioning, hydrogen peroxide, OH molecules can be produced as by-products of the hydroxylation reaction [31]. Probably, the mechanism of CYP102A1 (WT) radiation is due to the fact that the water-enzyme medium has a domain structure, which is nonequilibrium in the sense of the spin ratio between the ortho- and para-isomers of water, as indicated in the works [34,36]; upon excitation of an aqueous medium, a transition of the nonequilibrium state of the excited medium to an equilibrium state in the spin sense can occur, while radiation can be generated in the microwave range. The appearance of a train of microwave pulses of radiation can occur due to an enzymatic reaction. This causes both mechanical excitation of the medium due to the dropping and the appearance of an ice-like shell (water coat) in the surrounding enzyme molecules in different parts (domains) of the water-protein medium, and due to the appearance of excited OH molecules during the enzymatic reaction, which can emit in the microwave range [37] and initiate the emission of other OH molecules. This radiation can be realized as a cooperative process. The disappearance of the microwave radiation in the case of CYP102A1 (A264K) is possibly connected with the decrease in the amplitude of fluctuations of the mutant enzyme’s globule. In order to find out the influence of the A264K point mutation on the fluctuations of the CYP102A1 enzyme’s globule, we have conducted additional experiments on the AFM analysis of the functional activity of the enzyme.

As a result of the approximation (performed using Equation (3)), it has been obtained that the range of heights of the visualized CYP102A1 molecules both (WT) and (A264K) was 1.3 ÷ 6.5 nm, i.e., among them there were both monomers of a small height *h_max_*_1_ = (2.7 ± 0.1) nm, and objects of greater height *h_max_*_2_ = (3.5 ± 0.3) nm. The latter were attributed to oligomers according to the classification of objects with heights based on AFM images of CYP102A1 [30].

As is known, aggregates of CYP102A1 molecules are the most active functionally [28]. The heights of AFM images of both (WT) and (A264K) on mica amounted to 3.5 ± 0.3 nm. This is why, in our experiments, we studied the height fluctuations of CYP102A1—both (WT) and (A264K)—with (3.5 ± 0.1) nm height.

It was found that in the inactive state (in the absence of NADPH), the height fluctuation (∆*h*(*t*)) of the immobilized CYP102A1 (both (WT) and (A264K)) oligomers in the absence, and in the presence of the substrate (lauric acid) was the same. That is, the presence of the substrate did not affect the height fluctuation. The rms value of the heights of both (WT) and (A264K) oligomers in the presence of the substrate (lauric acid), averaged over 5 molecules, was 0.4 ± 0.1 Å. 

**Figure 5:**
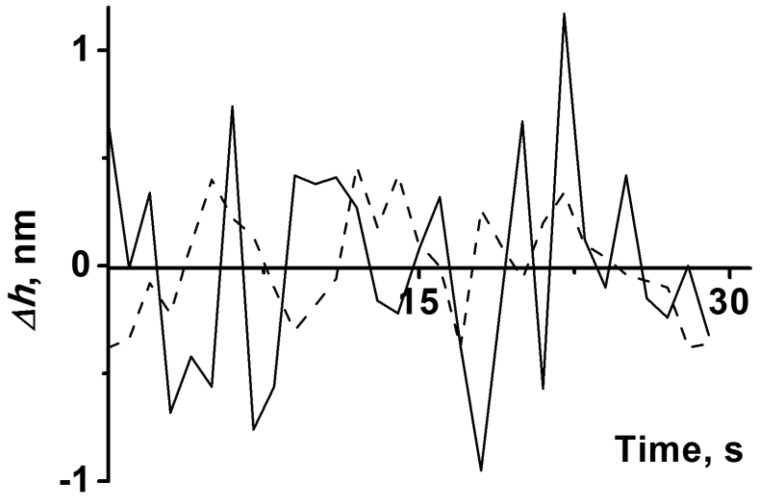
Typical time dependences of height fluctuations (∆*h*(*t*)) of the CYP102A1 (WT) enzyme globule in the presence of the substrate before (dashed line) and after (solid line) the addition of NADPH at the respective stages of its catalytic cycle. The protein oligomers’ height was (3.8 ± 0.1) nm. The AFM measurements in the presence of NADPH started from 5th minute of the enzyme’s catalytic cycle.

Figure 5 displays the typical time dependencies of height fluctuations (∆*h*(*t*)) of the globule of the CYP102A1 (WT) enzyme in the presence of the substrate before and after the addition of NADPH. Namely, the dashed line indicates the time dependence of the height fluctuations before the addition of NADPH, while the ∆*h*(*t*) dependence in the presence of NADPH is shown as a solid line. Under the conditions of hydroxylation, i.e., upon the addition of the NADPH electron donor to the incubation medium with CYP102A1 (WT), an increase in the amplitude of the enzyme globule oscillation to 0.8 ± 0.1 Å was observed, compared to the case without NADPH (Figure 5). Since a certain time was required for the preparation of the AFM measurements, we were able to perform the measurements starting from only the 5th minute of the enzyme’s catalytic cycle.

For CYP102A1 (A264K), analogous measurements have indicated that the amplitude of the enzyme globule oscillation upon adding NADPH was 0.6 ± 0.1 Å, exhibiting a tendency to decrease in comparison with CYP102 A1 (WT) during the enzyme functioning, though a tendency to the increase in the fluctuations of the mutant’s globule in comparison with its inactive state also takes place. This can mean the following. Upon the determination of the basic level of microwave radiation for the wild-type enzymatic system, we observed characteristic pulses of microwave radiation that appear when at the CYP102 A1 (WT) concentration of ~10^−9^ M. When the wild type of CYP102 A1 was replaced with the type A264K at the same enzyme concentration (C = 10^−9^ M) the radiation disappeared, indicating that during its functioning, the mutant enzyme type (8333-times less active) probably does not accumulate a sufficient quantity of ortho-isomers H_2_O and activated OH groups which could, in a water medium unbalanced in terms of spin distance, lead to the emergence of such a radiation as was detected for the wild type enzyme in [9]. Previously, at an 8333-times lower concentration of CYP102 A1 (WT), no microwave radiation was observed during the functioning of this system [38]; this fact can be explained by a decrease in the number of activated OH groups and H_2_O ortho-isomers. 

It should be emphasized that, according to AFM data, the difference between the enzymatic activity, registered by AFM in the form of fluctuations of the enzyme globule for the wild type enzyme (0.8 ± 0.1 Å) and for the A264K mutant (0.6 ± 0.1 Å), is not as significant in comparison with the 8333-fold difference in the activity obtained by spectroscopy measurements [28,29]. In [28,29], for the enzyme activity measurements, an approach, based on the control of NADPH oxidation at 340 nm, was employed. However, again, these authors have employed an optical macroscopic method, in which the signal form of an ensemble containing a large number of enzyme molecules was measured as opposed to AFM single-molecule measurements. In addition, in our opinion, this difference between the results, obtained by spectroscopic methods and by AFM, can be explained by the following. During the AFM measurements of enzymatic activity (by measuring the enzyme height fluctuations in tapping mode), the action of the AFM tip forces the structure of the globule of the mutant to optimize in order to provide optimal electron transfer. That is, in our AFM experiments, we have observed a nanomechanically assisted excitation of the mutant form of the enzyme. There can be, however, other reasons for occurrence of this phenomenon. For instance, the cooperative effect observed in aqueous medium during the fluctuations of the enzyme globule.

It should be noted that at low enzyme concentrations (10^−9^ M), microwave radiation appears in the form of individual pulses with a certain delay time required to initiate this process. It is known [38] that with an increase in the concentration of the CYP102A1 (WT) enzyme up to 10^−6^ M, the time of radiation pulse appearing after mechanical excitation decreases, and the intensity of pulses increases, while their number decreases to a single pulse. This means that, at high concentrations, synchronization of the time of occurrence of the microwave pulses and the solution stirring is observed owing to a cooperative process caused, among other things, by the microwave radiation from emitting molecules, acting as the impulse for the radiation of other excited molecules, while there is no linear dependence of the released energy in the process of enzyme functioning and concentration. The absence of such a linear dependence means that the process of microwave radiation is accompanied not only by the production of active forms of OH (which appear during the functioning of CYP102A1 (WT) and can emit in the microwave range), but also by radiation quenching due to absorption by inactive forms of OH, and other possible processes of nonspecific interaction of excited OH with the microenvironment. The complex radiation process of the CYP102A1 enzyme system is determined by several factors, such as viscosity, which inhibits the ortho/para transition of water isomers, and the process caused by the radiation of the domains of the water-protein medium associated with an increase in the number of functioning enzyme molecules (radiation factor).

For the studied mutant form of CYP102A1, the low rates of decomposition and the subsequent formation of the water shell around the enzyme globule can be explained by the 8333-fold reduced catalytic activity of the mutant enzyme, the form and reduced amplitude of the protein globule vibration compared to the wild type. This is the possible reason for the absence of microwave radiation for this mutant form at a concentration of 10^−9^ M.

The advantage of using the RTM method for studying the influence of mutations on enzyme kinetics—in comparison with the calorimetry method and the spectral methods—consists of that the RTM does not require expensive equipment, and the implementation of the RTM measurements is simple. Regarding the spectral methods, expensive optical systems are commonly utilized. In addition, the use of the RTM method does not eliminate the use of calorimetric, optical, and other methods, but RTM can be used as an additional method, in which quantum-mechanical mechanisms for the implementation of enzymatic processes are considered.

It should be noted that the RTM method is beginning to find its use in expression diagnostics of oncological diseases as a non-invasive method, which is very convenient for patients. In this work, we showed that enzyme mutations can lead to changes in the radiation characteristics of enzyme systems. Since mutant forms of proteins can be expressed in cancer diseases and their degradation occurs, these processes can be accompanied by a change in *T_br_* depending on the type of protein and its mutation. Therefore, the result of this work can be the basis for the development of new diagnostic systems based on RTM methods and analysis of the pathological state of the body caused by oncology at an early stage.

## 5. Conclusions

The applicability of the RTM method for the revelation of a point mutation in the enzyme on the activity of the enzyme system has been studied with the example of CYP102 A1. We have detected that generation of microwave radiation accompanying the reaction of hydroxylation by a nanoscale heme-containing enzyme CYP102 A1 that participates in metabolism of fatty acids, depends on a mutation that involves the enzyme’s functional properties. We know than amino acid replacements in the CYP102 A1 (A264K) enzyme led to an 8333-fold decrease in activity. The presented study shows that such a replacement of an amino acid leads to the disappearance of microwave generation. This allows the further use of microwave radiation monitoring to analyze possible point mutations in a molecule of this and other enzymes. The detected effect can be used to monitor the activity of enzymatic systems in order to create new systems to monitor the functional state of an organism, new systems of non-invasive diagnostics of oncological diseases connected with enzyme mutations.

## Data Availability

Correspondence and requests for materials should be addressed to Y.D.I.
